# The Wnt Pathway in Mood Disorders

**DOI:** 10.2174/157015912803217279

**Published:** 2012-09

**Authors:** Gabriele Sani, Flavia Napoletano, Alberto Maria Forte, Giorgio D Kotzalidis, Isabella Panaccione, Giulio Maria Porfiri, Alessio Simonetti, Matteo Caloro, Nicoletta Girardi, Carla Ludovica Telesforo, Giulia Serra, Silvia Romano, Giovanni Manfredi, Valeria Savoja, Stefano Maria Tamorri, Alexia E Koukopoulos, Daniele Serata, Chiara Rapinesi, Antonio Del Casale, Ferdinando Nicoletti, Paolo Girardi

**Affiliations:** 1 NESMOS Department (Neuroscience, Mental Health, and Sensory Organs), Sapienza University, School of Medicine and Psychology, Sant’Andrea Hospital, Rome, Italy; 2 Centro Lucio Bini, Rome, Italy; 3 NEUROMED, Pozzilli, Isernia, Italy; 4 Department of Neuropharmacology, Sapienza University, School of Medicine and Pharmacy, Rome, Italy; 5 Department of Neuropsychiatry, Villa Rosa, Suore Hospitaliere of the Sacred Heart of Jesus, Viterbo, Italy

**Keywords:** Wingless (Wnt) signalling, Mood Disorders, Bipolar Disorder, Major Depression, Antidepressant Drugs, Mood Stabilising Agents, Antipsychotic Drugs.

## Abstract

**Objectives:**

To review the evidence of the involvement of the Wnt signalling pathway in mood disorders and in the action of drugs used to treat these disorders.

**Methods:**

We performed a careful PubMed search using as keywords all possible terms relevant to the Wnt pathway and crossing them with each of four areas, i.e., developmental effects, behavioural effects, mood disorders, and drugs used in their treatment. Papers were selected on the basis of their content and their data used for discussion.

**Results:**

Neurodevelopmental and behavioural data point to the possibility of involvement of the Wnt pathway in the pathophysiology of mood disorders. Clinical and post-mortem data are not sufficient to corroborate a definite role for Wnt alterations in any mood disorder. Combining genetic and pharmacological data, we may state that glycogen synthase kinase is the key molecule in bipolar disorder, as it is connected with many other signalling pathways that were shown to be involved in mood disorders, while Wnt molecules in the hippocampus appear to be mainly involved in depressive disorders.

**Conclusions:**

Altered Wnt signalling may play a role in the pathophysiology of mood disorders, although not a central one. It is premature to draw conclusions regarding the possible usefulness of Wnt manipulations in the treatment of mood disorders.

## 1 INTRODUCTION

The Wnt signalling pathway or armadillo chain owes its name to *wingless* and Int-1, two proto-oncogenes that produce wingless mutations in *Drosophila* [1]. Wnt glycoproteins regulate morphogenesis in the developing central nervous system (CNS), and can also affect neuronal functions in adulthood [2,3]. In addition, abnormalities of the Wnt signalling pathway have been implicated in the pathophysiology of CNS disorders [see 4, for a review].

To date, there is no evidence that abnormalities in the Wnt signalling pathway are causally related to any psychiatric disorder; however, alterations of individual components of the pathway have been found in psychiatric disorders. The evidence that β-catenin levels are reduced in the hippocampal CA3 and CA4 regions, and Wnt1 levels are increased in the hippocampal CA4 region of post-mortem schizophrenic brains [5,6] fostered interest into the role of the Wnt signalling pathway in psychiatric disorders. Because lithium inhibits glycogen synthase kinase3β (GSK3β [7,8], a component of the canonical Wnt pathway (see below), the pathway has been proposed as a specific target in the treatment of bipolar disorders [9-12]. Here, we discuss (i) the physiological role of the Wnt pathway; (ii) preclinical data that suggest an involvement of the pathway in the pathophysiology of mood disorders (ii); the evidence for alterations in Wnt signalling in mood disorders; and (iv) the modulation of the Wnt pathway by drugs that are currently used in the treatment of mood disorders. 

## 2 METHODS

We searched the PubMed database using different strategies according to the section of the review. We used a general set of key words with all expressions denoting the pathway itself or the molecules known to be components of the Wnt signalling pathway (e.g., Wnt*, or wingless, or armadillo, or Dvl, or Dishevelled, or axin, or catenin*, or glycogen synthase kinase, or Frizzled, or Dickkopf*, or LRP5/6 receptors etc.). This set we used in all individual searches. To review the role of the Wnt pathway in human physiology and normal development, we crossed the above set with other key words, like neurodevelopment, development, apoptosis, cellular death, proliferation, oncogenesis, physiology, subsequently restricting to human studies. To gather behavioural studies of the Wnt pathway, we used the same strategy as above regarding the pathway, and crossed with general terms like behaviour and behavioural, specific terms like depression, psychosis, anxiety, hyperactivity, exploratory, immobility, avoidance, aggression, aggressiveness, aggressive, sedation, sniffing, grooming and the like, and paradigm-related terms, like forced swim test, tail suspension, open-field, marble burying etc. To identify studies dealing with the involvement of the Wnt pathway in mood disorders, we crossed the above set with another set containing terms like bipolar, depression, depressive, dysthymia or dysthymic, mixed state, agitation, mania, manic, rapid cycling, hyperthymic, dysphoric or dysphoria, suicide, suicidality, killed and self, and mood. Finally, to explore the effects of drugs used in the mood disorders on the Wnt pathway, we crossed the general set with subsets composed of keywords, like mood stabilisers or each one of them (i.e., lithium, carbamazepine, valproic acid or valproate or divalproate, oxcarbazepine, and lamotrigine, as well as the benzodiazepine clonazepam), antipsychotics or each antipsychotic, and antidepressants or each antidepressant. We then selected retrieved articles according to their relevance and further searched their References.

## 3 THE WNT PATHWAY IN HUMAN PHYSIOLOGY AND NORMAL DEVELOPMENT

The Wnt family in humans consists of 19 secreted proteins, termed Wnt1, Wnt2, Wnt2B, Wnt3, Wnt3A, Wnt4, Wnt5A, Wnt5B, Wnt6, Wnt7A, Wnt7B, Wnt8A, Wnt8B, Wnt9A, Wnt9B, Wnt10A, Wnt10B, Wnt11, and Wnt16. All these proteins undergo post-translational modifications and are 350-400 amino acid-long.

Wnt ligands interact with a number of surface receptors, which include Frizzled (Fzd) receptors, the low-density lipoprotein receptor-related protein 5 and -6 (LRP5/6), and the tyrosine kinase receptors, Ryk and Ror [13-15].

**Fig. (1):**
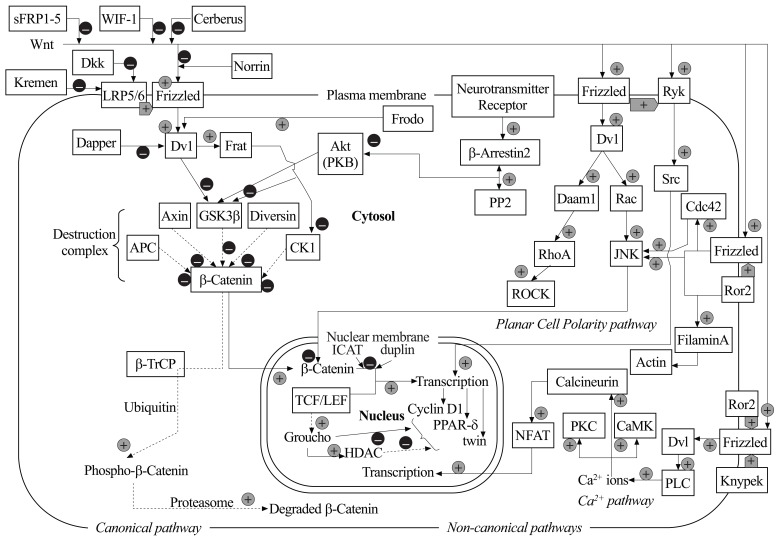
Schematic drawing of Wnt-related pathways. The canonical pathway depends always on β-catenin, whereas the other, non-canonical pathways may interact with β-catenin–dependent processes, but do not depend on the presence of β-catenin. Different Wnts may have different effects on overall cellular function, depending on the receptors involved and on the availability of co-receptors (LRP, Knypek, Ror2, Ryk etc.) Depicted are also some molecules not strictly belonging to the Wnt pathways, but having a central role in switching on or off β-catenin–regulating steps. Solid arrows in the canonical pathway show events with Wnt stimulating Frizzled; dashed arrows show events occurring in the absence of Wnt. Black arrows for the canonical pathway, grey arrows for the non-canonical; CaMK, calmodulin kinase; CK1, Casein Kinase 1; Dkk, Dickkopf; HDAC, histone deacetylase; Daam1, Disheveled-associated activator of morphogenesis 1; ICAT, Inhibitor of β-catenin and T-cell factor; JNK, Jun kinase; PKB, protein kinase B or Akt; PKC, protein kinase C; PLC, phospholipase Cβ; PP2, protein phosphatase 2A; PPAR-δ, Peroxisome proliferator-activated receptor delta; ROCK, Rho-associated kinase; sFRP1-5, secreted Frizzled-related proteins 1-5; β-TrCP, β-Transducin repeat Containing Protein; WIF-1, Wnt-inhibiting factor 1; +, enhancement, activation; –, down-regulation, inhibition.

Wnt signalling pathways are classified as β-catenin-dependent and β-catenin-independent, also known as canonical and non-canonical Wnt pathways, respectively (Fig. **1**). β-Catenin is an intracellular protein that exert pleiotropic functions, including adherent junction formation and gene expression regulation (see below).

In the “canonical” pathway, Wnt binds to Fzd and LRP5/6, thereby recruiting the scaffold protein, Dishevelled (Dvl), to the ligand-receptor complex. Activated Dvl binds to, and destabilises, a “β-catenin-destruction complex”, composed by GSK3β, adenomatous poliposys coli (APC), and axin. Within the destruction complex, GSK3β phosphorylates β-catenin, which is then committed to proteasomal degradation [13]. It is noteworthy that this is not the only function of GSK3β. GSK3β is a constitutively active serine/threonine kinase that phosphorylates a wide range of substrates, thereby regulating fundamental processes such as protein synthesis, glycogen synthesis, and apoptotic cell death [16-18]. To what extent these additional mechanisms of GSK3β are regulated by Wnt signalling is unclear. Activation of the canonical Wnt pathway causes an accumulation of undegraded β-catenin in the cytoplasm. β-Catenin then translocates into the nucleus, where it combines with the T-cell factor/lymphoid enhancer factor (TCF/LEF) family of transcription factors to regulate gene expression [13-19]. GSK3β is a point of convergence of multiple signalling pathways, including the phosphatidylinositol-3-kinase (PI3K)/Akt pathway, which is activated by a variety of G-protein coupled receptors and tyrosine kinase receptors, including the insulin receptor [20]. Phospho(activated)-Akt inhibits GSK3β by phosphorylating a serine residue (Ser^9^) in the N-terminus domain of the enzyme. The precise details of Wnt inhibition of GSK3β are unclear. One should bear in mind that the canonical Wnt pathway prevents the phosphorylation of β-catenin by GSK3β, but could leave other GSK3β functions intact. However, studies in Wnt overexpressing PC12 cells show that (i) activated Dvl recruits protein kinase B (Akt) at the β-catenin destruction complex; (ii) Akt inhibits β-catenin degradation by phosphorylating GSK3β on Ser^9^; and (iii) a negative dominant mutant of Akt decreases free β-catenin levels [21].

Some Wnt isotypes, including Wnt4, Wnt5a, and Wnt11, are able to activate “non-canonical” β-catenin-independent pathways, such as the Planar Cell Polarity (PCP) and Wnt/Ca^2+^ pathways [13,22] (Fig. **1**). In the Wnt/Ca^2+^ pathway, Wnt5a signalling through Fzd and Dvl activates phospholipase-Cβ, which stimulates the hydrolysis of phosphatidylinositol-4,5-bisphosphate with ensuing formation of the second messengers, inositol-1,4,5-trisphosphate (InsP_3_) and diacylglycerol (DAG). InsP_3_ stimulates Ca^2+^ release from intracellular stores, whereas DAG facilitates the activation of protein kinase C (PKC). PKC in turn activates Cdc42 (cell division control protein 42), a regulator of cell adhesion, cell migration and tissue pattering. The increase in intracellular Ca^2+^ activates a mitogen-activated protein kinase (MAPK) pathway composed of the transforming growth factor-β-activated protein kinase (TAK1), which is a MAPK kinase kinase, and the nemo-like kinase (NLK), which is a MAPK. This pathway negatively regulates the canonical Wnt pathway by inhibiting gene transcription mediated by the β-catenin/TCF complex [13,23-26]. In the PCP pathway, Wnt-mediated activation of Fzd and Dvl promotes the formation of the scaffold protein-Daam1 complex, which in turn activates the G-protein Rho. Rho activates ROCK (Rho-associated kinase), which is a key regulator of cytoskeleton dynamics [17,26,27]. The PCP pathway contributes to major developmental processes, such as body axis elongation and neural tube formation [28,29].

## 4 WNT IN NEURODEVELOPMENT

There are four major classes of secreted extracellular signalling molecules which are expressed in the developing brain during embryogenesis and participate in the patterning of the nervous system: Wnts, fibroblast growth factors (FGFs), Sonic Hedgehog (SHH), and Bone Morphogenetic Proteins (BMPs) [30]. Wnts, like BMPs, stem from the cortical hem, comprising the medial margin of each hemisphere. Wnt signalling has a recognized function in neurodevelopment since the early report of cerebellar and midbrain atrophy in Wnt1 knockout mice [31]. Regional specification of the developing brain depends on an anteroposterior gradient of Wnt signalling, being low for anterior structures and high for posterior structures [32,33]. Wnt signalling is also involved in processes of morphogenesis, neuronal migration and specification, and proliferation of neuronal progenitors [5,34]. In addition, a large body of evidence suggests a role for Wnt signalling in axon guidance, neurite outgrowth, and synaptic plasticity, including the regulation of long-term potentiation (LTP) of excitatory synaptic transmission in the adult brain [reviewed in 16]. Last, the Wnt signalling pathway contributes to the physiological cross-talk between neurons and astrocytes; disrupting this specific function may lead to neuro-degeneration in age-related disorders, such as Alzheimer's disease [2,35].

## 5 THE IMPORTANCE OF NEURODEVELOPMENT IN PSYCHIATRIC DISORDERS

The pathophysiology of most psychiatric disorders is still unclear. Recent data indicate a neurodevelopmental origin for schizophrenia [36-38], bipolar disorder [39], attention deficit/hyperactivity disorder [40], and autism and mental retardation [38,41,42]. Several studies suggest a role for Wnt-related proteins in the aetiology of autism, schizophrenia [4,34,43,44], and mood disorders [10,45].

## 6 BEHAVIOURAL STUDIES AND THE WNT PATHWAY

Recent data show that GSK3 regulates mood-related behaviour. The expression levels of GSK3β are associated with depression- and anxiety-like behaviours, as shown by an increased exploratory activity [46], reduced amphetamine-induced hyperactivity, and reduced immobility in the forced swim test [47] in mice lacking one copy of the GSK3β gene. However, GSK3β-overexpressing mice also show a reduced immobility in the forced swim test, a phenomenon that has been associated with a 20% reduction in the brain size [48]. Haploinsufficiency of the GSK3β gene also reduces depression-like behaviour in serotonin-deficient mice [49]; however, others found contrasting data [50]. An increased locomotor activity has been observed in mice expressing a constitutive active mutated form of GSK3β bearing a Ser to Ala substitution in position 9 [51]. Mice expressing an Akt-resistant form of GSK3β also display a reduced immobility time in the forced swim test, as well as a tendency to walk a longer distance in the unprotected area, and a higher number of unprotected head dips in the O-maze [52]. Recently, it has been shown that blockade of Dvl function and over-expression of GSK3β in the nucleus accumbens render mice more susceptible to social defeat stress and promote depression-like behaviour [53]. GSK3α knockin mice show decreased exploratory activity, decreased immobility time in the forced swim test, and reduced aggressive behaviour [54], suggesting that the two GSK3 isoforms have similar effects on behaviour. Accordingly, GSK3α/β knockin mice with serine-to-alanine mutations show increased susceptibility to amphetamine-induced hyperactivity and stress-induced depressive-like behavior, and serine-phosphorylation of GSK3 is reduced during mood-related behavioural responses [55]. These data raise the possibility that GSK3α and GSK3β have redundant functions in regulating mood-related behaviour [56-58]. As expected from the data of transgenic mice, pharmacological inhibition of GSK3 with lithium, NP031115, alsterpaullone, SB216763, L-803-mts and AR-A014418, decreases both the immobility time in the forced swim test, and amphetamine-induced hyperactivity [47,59-61].

Finally, GSK3 activity regulates circadian rhythms, with genetic depletion or inhibition of GSK3 resulting into a significant delay in the periodicity of the endogenous clock [62].

## 7 WNT AND MOOD DISORDERS

### 7.1 Bipolar Disorder Type I/II

Bipolar disorder (BP), also known as bipolar affective disorder or manic-depressive illness, is associated with alterations in synaptic formation and plasticity [63]. BP causes, in both childhood and adulthood, unusual shifts in mood, energy, and activity levels, from manic phases, characterised by euphoric mood, increased motor activity and energy, to phases of depression, characterised by loss of both energy and pragmatism. Manic episodes are characterised by periods of abnormally and persistently elevated mood (elation) or abnormal expansivity or irritability for at least one week (or needing hospitalisation), not due to substance use or medication or somatic antidepressant treatment. During these periods, affected persons show elevated self-esteem or grandiosity, increased energy and decreased need for sleep, early awakenings, pressured or racing thoughts, flight of ideas, loosening of associations, inappropriate talkativeness, goal-directedness, and involvement in immediately rewarding activities despite high probability of long-term damage. Behaviour during these periods may lead to social or occupational impairment or it may be characterised by psychotic symptoms. Major depressive episodes are characterised by depressed mood or loss of interest or pleasure in usually pleasurable activities, and extreme and opposite symptoms, like weight loss or gain, insomnia or hypersomnia, psychomotor agitation or retardation, or also decreased energy and fatigue, feelings of worthlessness or guilt, reduced ideational production, undecidedness, inability to concentrate or to store or retrieve memories, and recurrent thinking about death, although not necessarily about suicide and not simply fear of dying, and possible suicidal thoughts and plans. These symptoms must occur at least in a subset of five out of nine and in the same 2-week period (at least) and must not overlap with a manic episode. When the latter occurs, a mixed episode diagnosis is posed in the context of bipolar disorder. A hypomanic episode is similar to a manic one, save for the social-occupational impairment/psychotic symptom criterion. Major or unipolar depression is the occurrence of one or more major depressive episodes and is ruled-out when criteria for BP are met. BP is BPI when manic episodes occur and may alternate or co-occur (mixed episodes) with major depressive episodes; it is BPII when manic episodes have never occurred and hypomanic episodes alternate or co-occur with major depressive episodes. A BPII may become BPI and stay so, a BPI patient never becomes BPII [64].

#### 7.1.1 Animal Studies

Rats treated with the mood stabilisers, lithium and valproate, show increased levels of β-catenin, which is indicative of an activation of the Wnt pathway, in the frontal cortex [65]. GSK3β is inhibited by lithium, valproate, and antipsychotics by increasing GSK3β serine phosphorylation or directly inhibit enzyme activity [7,66-70]. A role for GSK3β in the pathophsiology of BP is suggested by the following observations: (i) mice overexpressing GSK3β show hyperactivity [51]; (ii) an increase in amphetamine-induced hyperactivity is associated with reduced serine-dephosphorylation of GSK3β [47]; and (iii) GSK3β inhibitors (as well as lithium) produce antidepressant-like effects [47,60,71].

#### 7.1.2 Human Studies

Post-mortem studies suggest that defects in neurodevelopment and neuroplasticity underlie BP. Grey matter volume is reduced by about 40% in the subgenual prefrontal cortex of BP patients [72] (Drevets *et al*., 1997), and this reduction is associated with glial cell loss and reduced neuronal number or size [73,74]. These findings prompted investigators to focus on Wnt-related molecules in post-mortem BP brain.

No changes in GSK3 expression were found in BP brains, compared healthy control brains [75,76]. Measurements of GSK3 activity in post-mortem human brain tissue are difficult because enzyme activity rapidly declines after death [77]. GSK3 activity can be assessed reliably in peripheral blood mononuclear cells (PBMCs) [78]. PBMC studies showed a significant decrease in serine phosphorylation of both GSK3α and GSK3β in symptomatic BP patients, as compared to healthy controls [55]. PBMCs were also used to assess GSK3 activity in response to drug treatment. Interestingly, phospho-Ser^9^-GSK3β levels were 8-fold in BP patients stabilized on lithium, compared to healthy controls [78]. These findings were confirmed in a group of patients with BPI who were hospitalized for a manic episode. Symptom improvement during an eight-week period of treatment with lithium, valproate, or atypical antipsychotics was associated with increased PBMC inhibitory GSK3β serine phosphorylation [79].

GSK3β gene studies have shown that some polymorphisms may underlie the susceptibility to mood disorders and may serve as potential markers of severity of such disorders [80]. Studying monozygotic twin pairs with one affected and one healthy member, four Wnt-related genes, KCNK1, MAL, PFN2, TCF7, and PGK1, were found to be overexpressed in BP [81]. Two SNPs (single nucleotide polymorphisms) in the promoter region of the GSK3β gene have been associated with BP; the –50T/C SNP and the C-variant polymorphism were found to be related to later onset, and to better response to acute sleep deprivation in BP patients [82-84]. The −1727A/T SNP has been associated with age at onset and presence of psychotic symptoms in BP [85]. In another study, the rs2267665 and rs9462082 SNPs in the PPARD (peroxisome proliferators-activated receptor type-δ) gene showed a significant association with BP, with rs9562082 being also related to symptom severity [86]. Studying the GSK3β locus, a copy number variation (CNV) in the 3' non-coding region, was found to be more frequent in BP patients than in healthy controls [87]. In a wider meta-analysis, the GSK3β locus was found to be implicated in BP by the widest array of studies, including association analysis, gene expression, pharmacogenomics, structural variants, and mouse models [88]. GSK3 mRNA levels were found to be lower in membranes and cytosolic fractions of platelets from drug-free patients with bipolar mania than in healthy controls or drug-free patients with major depressive disorder (MDD) [89]. Treatment with mood stabilisers restored GSK3 mRNA levels in BP-I patients, while antidepressant treatment did not affect GSK3 mRNA levels in platelets of patients with MDD [89].

Whether changes in the genes encoding for other Wnt-related molecules are associated with BP remains to be established. Four polymorphisms in the FZD3 gene (rs960914, rs2241802, rs2323019, and rs352203) have not been associated with BP [90].

### 7.2 Major Depressive Disorder (MDD)

As in bipolar disorder, abnormalities in neuronal plasticity and neurogenesis strongly concur to the pathogenesis of MDD. Recent studies suggest an important role for Wnt signalling pathway in MDD.

Different Wnt isotypes, including Wnt2, 3a, and 7a, are expressed in the rat hippocampus, where an increased Wnt2 expression was shown after chronic treatment with various classes of antidepressants [91] or after chronic electro-convulsive seizures [92]. In addition, viral-induced overexpression of Wnt2 in the hippocampal dentate gyrus produced a clear-cur antidepressant effects using behavioural paradigms endowed with pharmacological validity as tests for antidepressant medication [91]. Furthermore, Wnt3a contributes to hippocampal neurogenesis [14] and Wnt7a promotes synaptogenesis, dendrite maturation and an increase in excitatory synapses [93,94].

Along the Wnt signalling pathway, various components have been specifically implicated in physiological neurogenesis and its anomalies, like Frizzled receptors, Dvl, and GSK3β and β-catenin.

Regarding the Frizzled receptor family, Fdz6 is a seven transmembrane-spanning receptor whose Wnt binding regulates activation of canonical and non-canonical pathways, specifically Ca^2+^- and PCP-pathways [95]. Fdz6 knockdown models have been studied to better understand behavioural changes; knockdown was related to decreased sucrose preference, which is assumed to represent an anhedonic response, and to increased anxiety in the suppressed feeding test and in the elevated plus maze (EPM); similar data were obtained also through chronic unpredictable stress (CUS) exposure. CUS has also shown decreased Fzd6 expression, suggesting that Fzd6 modulation can concur to the behavioural effects of CUS [96,97]. These results show a specific action of Fdz6 on anhedonia and anxiety, but not on despair, with decreased escapes in the learned helplessness test [96,97]. The way Fdz6 regulates these behaviours is still unknown.

Dvl and β-catenin play a critical role in dendritic arborisation [98] and in axon differentiation, respectively. Concerning GSK3β, its direct inhibition shows an antidepressant-like activity [60]. On the other side, GSK3β is inhibited by antidepressants with serotonergic activity [99].

Genetic studies have thoroughly investigated the role of GSK3β polymorphisms and genes involved in Wnt signalling in the cerebral cortex. These studies shed light on the association between polymorphisms and various regional changes in brain structure observed in MDD patients [100,101]. Several lines of evidence implicate such polymorphisms in vulnerability for mood disorders [80,100,101].

Regional grey matter (GM) volume changes in MDD patients were found in temporolateral and medial prefrontal cortices, in association with SNPs in canonical Wnt signalling pathway genes and in GSK3β substrate proteins genes [100]. GM volume changes in the right hippocampus and bilateral superior temporal gyri are strictly associated with a specific SNP, –157T/C, an intronic polymorphism that regulates the selection of splice acceptor sites of GSK3β, influencing GSK3β transcription [101].

Other four SNPs in the non-coding region of the GSK3β, –50 T/C, +9227 A/G, +11660 G/T and +4251 T/A have been significantly associated with 4-week antidepressant response [102].

Post-mortem studies showed increased activity of GSK3β in suicide victims with MDD [103] and abnormal GSK3β activity in patients with MDD [55]. A study of GSK3β mRNA expression showed an increase in the hippocampus of MDD patients compared with healthy controls [104]. These post-mortem studies should be interpreted with caution for the same reasons we explained in the bipolar disorder section.

Combining these data we may state that Fzd receptors play a role in neurogenesis, synaptic and neuronal plasticity. Thus, it is possible that they are involved in the pathogenesis of MDD. Genetic studies heretofore identified polymorphisms of genes encoding interacting proteins in brain regions involved in MDD, such as hippocampus and temporal gyrus. Genetic variations along the Wnt pathway may be not only involved in disease vulnerability, but also in treatment response. These data need replication.

### 7.3 Suicide

Suicidal ideation occurs more frequently in mood disorders and death by self-killing is more frequently associated with depression, a state common to BP and MDD and the major source of burden in both disorders.

The Wnt signalling pathway in suicide has not been widely studied so far. Recent post-mortem studies showed increased GSK3β and decreased Akt activities in brains of suicide victims with MDD, but not in suicide victims without depression [103].

One genetic study tackled the issue of genetic polymorphisms and suicidality, focusing on polymorphisms of two common GSK3β SNPs, –1727A/T and –50C/T [105]. Findings showed that SNP alleles, genotypes, and haplotypes did not differ between suicidal MDD patients, non-suicidal MDD patients and healthy controls. Another study was a genoma-wide association study in a suicide attempt bipolar population [106]. This study compared SNP genotypes of more than 1000 bipolar suicide attempters with those of a comparable bipolar population without suicide attempt history. Investigators identified 2507 SNPs with evidence for association at *P*<0.001; however, when these SNPs were subsequently tested for association in another large and independent bipolar sample, no one was found to be significantly associated after correcting for multiple testing. Despite this, the combined analysis of all sample sets produced an association signal at the threshold of genome-wide significance only for 2p25 (rs300774), with the associated SNPs on 2p25 falling in a large linkage disequilibrium block containing the ACP1 (acid phosphatase 1) gene, a gene whose expression was significantly elevated in people with bipolar disorder who committed suicide. The ACP1 protein is a Wnt signalling influencing tyrosine phosphatase.

## 8 THE EFFECTS OF DRUGS USED IN MOOD DISORDERS ON THE WNT SIGNALLING PATHWAY 

### 8.1 Mood Stabilisers

#### 8.1.1 Lithium

Lithium is the gold standard in the treatment of BP. It is widely used in the treatment of acute manic episodes and in the prophylaxis of manic and depressive episodes. In addition, lithium possesses an independent antisuicidal action [107]. Lithium has multiple mechanisms of action in neurons, which include the modulation of ion channels and membrane transporters, and the inhibition of intracellular enzymes such as inositol monophosphate phosphohydrolase [108,109], phosphoglucomutase [19], protein kinase C (PKC), and GSK3β [7]. Lithium protects neurons against a variety of insults [110-116], an effect that has been attributed to GSK3 inhibition [7,12,89,107,117].

Klein and Melton [7] and Hedgepeth *et al*., [8] showed for the first time that “therapeutic” concentrations of lithium could inhibit GSK3, with ensuing activation of β-catenin-mediated gene transcription. This suggested that (i) GSK3β is involved in the pathophysiology of BP; and (ii) lithium interferes with the canonical Wnt pathway. Lithium inhibits GSK3 through direct and indirect mechanisms. Lithium directly inhibits GSK3β through competition for a low affinity Mg^2+^ binding site [19]. The indirect mechanism is mediated by the activation of enzymes that phosphorylate a serine residue at the N-terminal domain of GSK3β, consequently inhibiting enzyme activity. This action of lithium was shown in PBMCs [78], cultured cells [118], and mouse brain [67]. How lithium increases serine phosphorylation of GSK3β is not entirely clear. The disruption of a β-arrestin/Akt/protein phosphatase-2A (with resultant Akt activation) or indirect protein phosphatase 1 inhibition were advanced to explain it [118-120]. Thus, the synergistic effects of multiple mechanisms may account for the substantial inhibition of GSK3β by lithium [121].

A series of genetic studies in an Italian population indicated that a SNP in the promoter region of GSK3β (–50T/C) was associated with BP, with the C-variant being associated with later onset and better response to lithium or acute sleep deprivation [82-84]. In an independent study, the C-carriers of the -50T/C SNP responded better to lithium augmentation in acutely depressed, antidepressant-resistant BP and MDD patients [122]; however, Szczepankiewicz *et al*., [123] did not find a significant association between the –50T/C SNP and GSK3β response to prophylactic lithium in BP. 

A role for GSK3β inhibition (and the ensuing activation of the canonical Wnt pathway) in the therapeutic action of lithium in mood disorders is supported by preclinical studies. Acute administration of lithium or valproate increases β-catenin levels in the rat frontal cortex [65]. Lithium treatment mimicked the effect of GSK3β inhibitors or the effect of loss-of-function mutations of GSK3β in reducing amphetamine-induced hyperactivity and depressive-like behaviour in rodents [47,59,60,124]. Selective GSK3β inhibitors and lithium attenuate locomotor hyperactivity in response to novelty in mice carrying mutations in the circadian gene, *Clock* [124]. *Clock*-mutant mice were advanced as a genetic mouse model for bipolar mania [125], and polymorphisms of the CLOCK gene have been associated with BP and other mood disorders [126-129] (but see also [130] and [131] for contrasting results). The administration of lithium with olanzapine mimicked the effect of GSK3β inhibitors in a new model of mania in male adult mice, as they both reduced amphetamine-induced rearing locomotion [132].

Lithium proved to reverse stress-induced alterations in spatial memory [133] and dendrite architecture in the hippocampus [134]. In addition, lithium treatment blocked stress-induced depressive-like behaviour and hippocampal cell fate, and prevented stress-induced changes in the expression of GSK3β and its target genes in the hippocampus [135]. This suggests that GSK3β is a nodal point in the processes of maladaptive neuronal plasticity triggered by chronic stress and is largely involved in the therapeutic effects of lithium in mood disorders.

#### 8.1.2 Other Mood Stabilisers: Valproate, Lamotrigine, Carbamazepine

The discovery that lithium acts as an inhibitor of GSK3 raised interest in examining whether other mood stabilisers are able to modulate GSK3 activity.

Valproate, a simple branched-chain fatty acid (2-propylpentanoic acid), is an antiepileptic drug with demonstrated efficacy in the treatment of BP-associated acute mania and in the prevention of manic episodes of BP. Valproate is sometimes used to reduce the risk for manic switch in patients with bipolar depression, when antidepressants are concurrently used [136], despite the drug lacks antidepressant effects of its own [137].

Whether valoproate acts as an inhibitor of GSK3β is matter of debate [66,138-143]. Valproate was shown to inhibit GSK3 through a variety of mechanisms, such as serine phosphorylation [66,68,139], *tau* protein phosphorylation-mediated GSK3 inhibition in human neuroblastoma cell lines overexpressing GSK3β [144], or through the action of metabolites that potently inhibit GSK3 activity *in vivo* [138]. If present, inhibition of GSK3β by valproate is seen at therapeutic concentrations [65]. Valproate is less potent than lithium in inhibiting GSK3β [68]; however, valproate and lithium increase serine phosphorylation *via* two different mechanisms, which results into a synergic enzyme inhibition when the two drugs are combined [145]. Valproate acts as a week inhibitor of histone deacetylases (HDACs), a mechanisms that is apparently not shared by lithium. HDAC inhibition enhances Akt phosphorylation, with ensuing inhibition of GSK3β [67,68,146,147]. In spite of this additional mechanism, valproate did not share the ability of lithium to inhibit GSK3β activity in cultured cortical neurons [148]. In cultured cerebellar granule cells lithium, but not valproate, inhibited GSK3β and was protective against apoptotic death [143]. In another study, however, both lithium and valproate were able to protect cultured granule cells against GSK3-mediated apoptosis [149]. Data of *in vivo* studies are not homogeneous. Kozlovsky *et al*., [150] showed that subchronic valproate treatment did not change GSK3β levels or activity in the adult rat frontal cortex. However, the same paradigm of valproate administration was found to increase the levels of Ser^9^ phosphorylated (inhibited)-GSK3β in the frontal cortex [68]. In another study, valproate administration attenuated hypoxia-induced dephosphorylation of brain GSK3α and GSK3β without altering total GSK3 protein content in mice [151].

The influence of valproate on the Wnt pathway is not restricted to the inhibition of GSK3β. Zhou *et al*., [152] have found that chronic treatment with valproate (or with lithium) induces changes in the hippocampal levels of the microRNAs, let-7b, let-7c, miR-128a, miR-30c, miR-221, and miR-144. Some of the mRNA targets for these miRNAs encode for proteins of the canonical Wnt pathway.

Human studies on valproate and the Wnt/GSK3β/β-catenin pathway have generated contrasting data. An eight-week treatment of BP patients with valproate, lithium, and/or atypical antipsychotics enhanced serine phosphorylation of GSK3 in peripheral blood mononuclear cells. Interestingly, concomitant electroconvulsive therapy prevented this effect [79]. Post-mortem studies did not show alterations in GSK3β and β-catenin levels [76,153,154], GSK3α and GSK3β mRNA levels, or total GSK3 activity [153] in the frontal cortex of BP patients. More importantly, no changes were found after treatment with mood stabilisers, including valproate [154].

Thus, whether and how precisely valproate influences Wnt signalling, and whether this contributes to the therapeutic efficacy of valproate in BPs, remains unclear.

Few studies have investigated the effect of treatment with antiepileptic drugs used as mood stabilisers, such as carbamazepine or lamotrigine, on GSK3β and other Wnt-related proteins. There is only an unpublished observation that chronic lamotrigine treatment increased serine phosphorylation of GSK3 in the mouse hippocampus or cerebral cortex (reported in [147]). Other studies consistently failed to show GSK3 inhibition by carbamazepine or lamotrigine [147,148,155] (Table **1**).

### 8.2 Antipsychotic Drugs

Antipsychotics are used in patients with schizophrenia, in the manic phase of BP, in other psychotic disorders, like delusional disorder or shared psychotic disorder, and in depression with psychotic features. Atypical antipsychotic drugs antagonize 5-HT_2A_ serotonin receptors, and, with the exception of risperidone and 9-hydroxy-risperidone, show a low D2 dopamine receptor occupancy and are therefore devoid of extrapyramidal side effects. It is generally believed that psychosis is associated with an increased activity of the meso-limbic dopaminergic system, and a reduced activity of the meso-cortical dopaminergic system [156-159]. Beaulieu *et al*., [47] showed that an increased dopaminergic transmission resulting from amphetamine administration or deletion of the high affinity dopamine transporter caused inactivation of Akt and concomitant activation of GSK3α and GSK3β in the striatum, an effect reversed by lithium or D_2_ receptor blockade. Others confirmed that dopaminergic drugs increase the activity of GSK3β [55,160], while antipsychotic drugs produce the opposite effect [69,161-165].

Clozapine, haloperidol, and risperidone were found to increase GSK3 phosphorylation, as well as Dvl-3 and β-catenin levels in the rat medial prefrontal cortex and striatum [166]. Levels of Dvl-3, GSK3, and β-catenin were also increased in the hippocampus and ventral midbrain following repeated antipsychotic treatment [167]. X. Li *et al.,* [69] found that acute treatment with atypical antipsychotics, such as clozapine, risperidone, olanzapine, quetiapine, and ziprasidone, increased Ser^9^ phosphorylation of GSK3 in the mouse cerebral cortex, hippocampus, striatum, and cerebellum. There was a synergism between risperidone and antidepressants (monoamine oxidase inhibitors or fluoxetine) in enhancing GSK3 phosphorylation, an evidence that supports the use of risperidone in mood disorders [69]. Chronic treatment with clozapine or haloperidol also increased levels of Wnt-5a (but not Wnt1 and Wnt3a), Axin, Dvl-3, total and Ser^9^-phosphorylated GSK3, and β-catenin in the frontal cortex [162]. As outlined above, GSK3β can be phosphorylated on Ser^9^ by multiple signalling pathways including the canonical Wnt pathway and the PI3K/Akt pathway. Roh *et al*., [168] found that clozapine and haloperidol differentially regulate signals upstream of GSK3 in the rat frontal cortex. Both drugs were able to phosphorylate GSK3α/β on Ser^9^ and Ser^21^; however, only clozapine increased Dvl phosphorylation. In neuroblastoma cell lines showed that clozapine increased Ser^9^ phosphorylation of GSK3β* via* the activation of the canonical Wnt pathway, but not *via* the activation of the PI3K/Akt pathway [161]. Whether, and to what extent, haloperidol inhibits GSK3 *via* the PI3K/Akt pathway is matter of debate [169-171]. On the basis of these findings, it is counterintuitive that spiperone, an antipsychotic agent structurally related to haloperidol *inhibits *Wnt signalling. This unexpected effect is secondary to increases in intracellular free Ca^2+^ which results from a thapsigargin-like activity of spiperone at intracellular Ca^2+^ stores [172]. This adds further complexity to the mechanisms whereby antipsychotic drugs regulate Wnt signalling (see also Beaulieu [173].

The modulation of Wnt signalling by antipsychotic drugs might have a strong impact on the alterations of neuronal connectivity and cell fate associated with psychotic disorders. In a recent elegant study, Brennand *et al*., [174] demonstrated that fibroblasts from schizophrenic patients reprogrammed into pluripotent stem cells and then differentiated into neurons show diminished connectivity and an altered expression of many components of the Wnt pathway, including Axin2, Wnt2B, Wnt3, Pik3R3, TCF4, LEF1, RAP2A, LRP5, and Wnt7A. Interestingly, these alterations are corrected by treatment with loxapine, but not with other antipsychotics. 

Taken together, these data suggest that antipsychotics share the ability of lithium and valproate to activate Wnt signalling through the inhibition of GSK3β or other mechanisms (Table **1**). This may underlie the antimanic and mood-stabilising activity of antipsychotics in BP.

### 8.3 Antidepressants

Few studies investigated the action of antidepressant drugs on the Wnt pathway.

GSK3 activity is regulated by 5-HT_2_ and D_2_ receptors in specific neurons [175]. Serotonergic and dopaminergic mechanisms are involved in the action of all antidepressant drug classes, from tricyclic antidepressants and monoamine oxidase inhibitors to selective serotonin reuptake inhibitors (SSRIs) and serotonin and noradrenaline reuptake inhibitors (SNRIs). Much like antipsychotics and mood stabilisers, monoamine-regulating antidepressant drugs inhibit GSK3. For example, both imipramine and fluoxetine and imipramine greatly increased inhibitory serine GSK3 phosphorylation in the mouse brain [49,99]. However, we should stress that antidepressant drugs inhibit GSK3 activity a few hours after a single administration, whereas their therapeutic efficacy in MDD requires 3-4 weeks of daily administration. Thus, we may consider GSK3 inhibition as an initial event leading to slowly developing neuroadaptation, which underlies the therapeutic efficacy of antidepressants on mood disorders. Interestingly, Wnt signalling activation has been associated with antidepressant-induced increased hippocampal neurogenesis [176]. Accordingly, fluoxetine promotes neurogenesis in the hippocampal dentate gyrus by up-regulating Wnt3a [177], whereas subchronic fluoxetine increases non-nuclear β-catenin expression [178] and chronic treatment with venlafaxine enhances both the proliferation of neuro-progenitor cells and nuclear translocation of β-catenin in the hippocampus [179]. Antidepressants may affect the function of the canonical Wnt pathway through manipulation of Akt-1 phosphorylation, which is enhanced by fluvoxamine, both acutely [180] and chronically [181], and inhibited by sertraline [182]. Imipramine, like fluvoxamine, increased phospho-Ser^473^ Akt level, but did not affect total Akt levels [183]. Generally, antidepressants increase neuroplasticity through Wnt-related pathways or through pathways that affect sideways the canonical Wnt pathway. However, each drug may have its own specific actions which may be clinically important and contrasting on specific functions; for example, fluvoxamine increases Akt-phosphorylation through sigma1-receptors [180,181], while sertraline decreases it [182].

## 9 CONCLUSIONS

Data suggesting a possible involvement of the Wnt signalling pathway in the pathophysiology of BP and MDD are sound, but not always consistent. We should underline that Wnt signalling has not been studied in other mood disorders, such as dysthymic disorder or mood disorder, not otherwise specified. The Wnt-related protein that has been consistently associated with BP or MDD is GSK3β; however, this molecule is at the crossroads between other signalling pathways that also appear to be involved in mood and psychotic disorders (see Fig. **1**). The relative contribution of the Wnt pathway and other signalling pathways to the dysregulation of GSK3β associated with BP or MDD remains to be established.

The action of some drugs used in mood disorders, particularly lithium, valproate, and antipsychotic drugs, involves the canonical Wnt pathway. These drugs regulate Wnt signalling through a number of mechanisms that are both drug-specific and brain region specific. Whether non-canonical Wnt pathways are also involved in the action of antipsychotic drugs is entirely unknown. Drugs that selectively activate the canonical Wnt pathway (e.g., selective GSK3 inhibitors) might improve mood disorders without the adverse effects associated with the use of the current antidepressants or mood stabilisers. However, the complexity of the mechanisms underlying the pathophysiology of mood disorders might favour the use of drugs with multiple mechanisms of action over the use of small molecule inhibitors of individual enzymes. This particular issue is critical to the design of new drugs in psychiatric disorders and warrants further investigation.

## Figures and Tables

**Table 1.** Wnt Pathway Molecules Involved in the Action of Mood Stabilisers, Antipsychotics, and Antidepressants

*Wnt Molecules*				
*Drugs*	GSK3 function	GSK3β Ser9 Phosphorylation Inactivation	AKT Phosphorylation	β-Catenin
Mood stabilisers				
Lithium	↓	↑	⬄	↑
Valproate	⬄	↑	↑	↑
Carbamazepine	⬄	⬄	n.d.	⬄
Lamotrigine	⬄	⬄↑	n.d.	n.d.
Antipsychotics				
Haloperidol	↑	↑	↑, transient	↑
Clozapine	↑	↑	↑	⬄-↑
Risperidone	↑	↑	⬄	↑
Olanzapine	n.d.	↑	n.d.	n.d.
Quetiapine	n.d.	↑	n.d.	n.d.
Ziprasidone	n.d.	↑	n.d.	n.d.
Antidepressants				
Imipramine	⬄	↑	↑	n.d.
Fluoxetine	n.d.	↑	n.d.	↑
Fluvoxamine	n.d.	n.d.	↑	↑
Sertraline	n.d.	n.d.	↓	n.d.
Venlafaxine	n.d.	n.d.	*	↑

↑, increase;

↓, decrease;

⬄, conflicting evidence or lack of effect; n.d., no data, not determined,

*, increases levels, but no data on phosphorylation.

## ABBREVIATIONS

ACP1: = Acid phosphatase 1
Akt: = Protein kinase B/Non-specific serine/threonine protein kinase family
APC: = Adenomatous poliposys coli
BP: = Bipolar disorder
BMPs: = Bone Morphogenetic Proteins
Ca^2+^: = Calcium ion
Cdc42: = Cell division control protein 42
CNV: = Copy number variation
CUS: = Chronic unpredictable stress
DAG: = Diacylglycerol
Dvl: = Dishevelled
EPM: = Elevated plus maze
FGF: = Fibroblast growth factor
FZD: = Frizzled
GM: = Grey matter
GSK3(α/β): = Glycogen synthase kinase-3(alpha, beta)
HDAC: = Histone deacetylase
InsP3: = Inositol-1,4,5-trisphosphate
LRP5/6: = Low Density Lipoprotein receptor-related protein 5 and -6
LTP: = Long-term potentiation
MAPK: = Mitogen-activated protein kinase
Mg^2+^: = magnesium ions
MDD: = Major Depressive Disorder
NLK: = Nemo-like kinase
PPARD: = Peroxisome proliferators-activated receptor type-δ
PBMC: = Peripheral blood mononuclear cell
PCP: = Planar Cell Polarity
PI3K: = Phosphatidylinositol-3-kinase
PKC: = Protein kinase C
ROCK: = Rho-associated kinase
SHH: = Sonic Hedgehog
SNP: = Single Nucleotide Polymorphism
TAK1: = Transforming growth factor-β-activated protein kinase
TCF/LEF: = T-cell factor/lymphoid enhancer factor family of transcription factors
Wnt: = Wingless
